# The Role of EPFL Peptides in Plant Development and Stress Responses

**DOI:** 10.32607/actanaturae.27675

**Published:** 2025

**Authors:** A. D. Maiboroda, A. A. Makeeva, R. A. Azarkina, A. S. Barashkova, A. S. Mamaeva

**Affiliations:** Shemyakin–Ovchinnikov Institute of Bioorganic Chemistry, Russian Academy of Sciences, Moscow, 117997 Russia; All-Russian Institute of Plant Protection, St. Petersburg, Pushkin, 196608 Russia

**Keywords:** plant regulatory peptides, cysteine-rich peptides, EPF/EPFL

## Abstract

Cysteine-rich peptides belonging to the EPF/EPFL (epidermal patterning factor/epidermal patterning factor-like) family are common in many plants, from mosses to angiosperms. EPF/EPFL play an important role in morphogenesis: they regulate stomatal patterning, the functioning of the shoot apical and lateral meristems, inflorescence architecture, vascular development, growth of leaf margin, as well as the development of flowers and fruits. Recent studies have indicated that EPFL may be involved in plant adaptation to biotic and abiotic stress. This review examines the structure, phylogenetic distribution, mechanisms of signal transduction, and functions of the EPF/EPFL peptide family.

## INTRODUCTION

As sessile organisms, plants adapt to environmental changes through a flexible system that regulates physiological processes. A crucial role in this adaptation is played by signal peptides, which control a broad range of responses, including growth and development, sexual reproduction, intercellular communication, senescence, symbiosis, as well as resistance to pathogens and abiotic stress [1, 2]. The first identified plant regulatory peptide, systemin, was isolated from tomato leaves in 1991 [3]. Numerous peptide families, originating either from the processing of precursor proteins or via translation of short open reading frames, have been described since then [1, 4].

Peptides derived from precursor proteins are classified into three functionally and structurally distinct groups: post-translationally modified peptides [5], cysteine-rich peptides, and unmodified peptides without cysteine residues [6, 7]. Cysteine-rich peptides carry an even number of cysteine residues that form disulfide bonds, a disposition that ensures the stability of their spatial structure. Antimicrobial peptides were the first members of this group to be discovered and described [8]. It was originally believed that the functions of cysteine-rich peptides were limited to defense against pathogens [4, 9]. However, subsequent research demonstrated that cysteine-rich peptides have a much broader range of functions, encompassing the regulation of stomatal initiation, symbiosis, reproductive processes, and stress responses [10, 11, 12].

The cysteine-rich peptides EPF/EPFL were first identified as key regulators of stomatal development in Arabidopsis thaliana (Arabidopsis) [10, 13, 14, 15]. Further research revealed that these peptides are involved in the regulation of the size of shoot apical meristem, inflorescence development, and stress adaptation. Although the body of experimental data on the subject continues to grow, there are currently no systematic reviews that summarize information about this family. Our study has endeavored to consolidate the data on EPF/EPFL peptides, including their structure, evolutionary diversity, and biological functions.

## THE STRUCTURE AND SIGNAL TRANSDUCTION

**Fig. 1:**
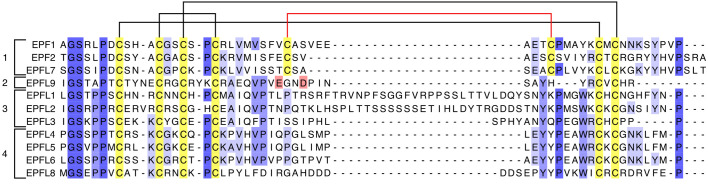
Multiple alignment of mature peptides belonging to the EPF/EPFL family in A. thaliana conducted using the Muscle algorithm. (1–4) clades of peptides; cysteine amino acid residues are highlighted in yellow. Three conservative disulfide bonds are indicated with black brackets; the fourth disulfide bond, specific to the EPF1/EPF2/EPFL7 clade, is indicated with red bracket. Glu28 and Asp31 amino acid residues in EPFL9 are highlighted in pink. UniProt ID: EPF1: Q8S8I4; EPF2: Q8LC53; EPFL7: C4B8C5; EPFL9: Q9SV72; EPFL1: Q9LFT5; EPFL2: Q9T068; EPFL3: C4B8C4; EPFL4: Q2V3I3; EPFL5: Q9LUH9; EPFL6: Q1PEY6; EPFL8: Q1G3V9

Cysteine-rich plant peptides can be roughly divided into defensive (antimicrobial) and regulatory peptides and comprise several families, including the EPF/EPFL one [16]. The structure of defensive peptides has been the one studied most thoroughly: NMR analyses have been performed for many of these peptides, and the structural determinants of their antimicrobial activity have been identified [17, 18]. The structural features of cysteine-rich signal peptides in plants, including the EPF/EPFL family, have been investigated less thoroughly; however, the primary and spatial structures of the EPFL9 peptide isolated from the A. thaliana apoplast have been determined [19, 20]. Furthermore, structural data on peptide-receptor interactions for several peptides belonging to this family has been obtained [21]. Eleven peptides have been identified in A. thaliana, the classical model organism that is most commonly used to study this peptide family: EPF1–2 and EPFL1–9, including EPFL9/ Stomagen [22]. EPF1 and EPF2 were the first to be characterized, followed by other EPF1 paralogs named EPFL [23]. The EPF/EPFL peptides were divided into four clades by phylogenetic analysis (Fig. 1). Members of two of these clades, EPF1–EPF2–EPFL7 and EPFL9, have been the most thoroughly studied.

**Fig. 2:**
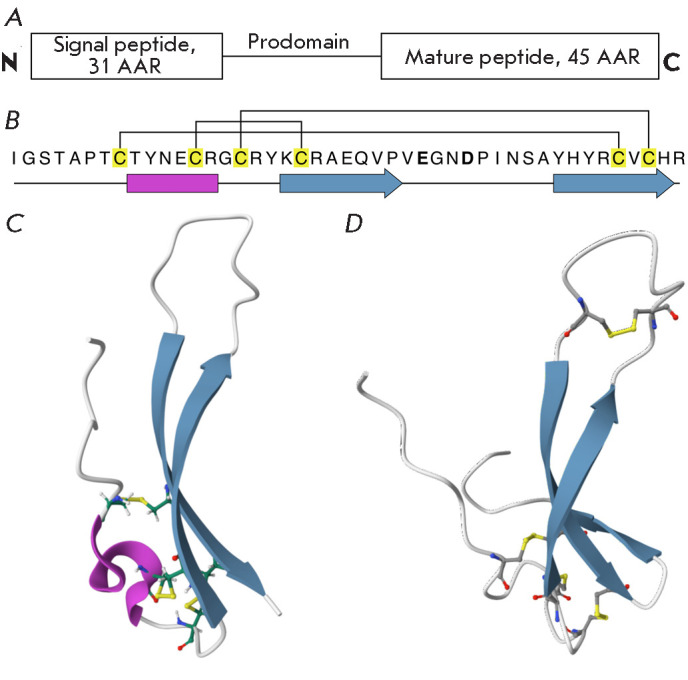
The structure of EPFL9 peptide. (A) The structure of preproprotein [20]. (B) The primary structure of peptide [19]. β-sheets are shown with blue arrows; the 3_10_-helix is shown with a pink rectangle; Cys residues are highlighted in yellow; disulfide bonds are shown with brackets. Negatively charged amino acid residues Glu28 and Asp31 in the loop region are highlighted in bold. (C) The spatial structure of EPFL9 (PDB ID: 2LIY). (D) The structural model of EPFL7 peptide in A. thaliana, generated using the AlphaFold3 algorithm [26]

Like most peptide hormones and antimicrobial peptides in plants, members of the EPF/EPFL family are synthesized as precursor proteins consisting of an N-terminal signal peptide, a prodomain, and a mature peptide (Fig. 2A) [24]. The signal peptide guides the precursor to the endoplasmic reticulum, where it is then cleaved off and degraded by peptidases. The prodomain is subsequently removed, and a mature peptide capable of interacting with receptor complexes is released [25].

The primary structure of EPF/EPFL peptides is rich in cysteine residues; six of them are conserved across the entire family, and two additional residues occur only in the EPF1/EPF2/EPFL7 clade (Fig. 1). All the peptides belonging to this family carry the Gly-Ser motif in the N-terminal region. This motif is known to be critical in peptides binding to their receptors [21]. A conserved Pro residue is also present in the N-terminal region. This residue probably helps maintain the spatial conformation of the peptide by bending the polypeptide chain.

The NMR spectroscopy data garnered for EPFL9 suggest that the three-dimensional structure of EPF/EPFL peptides consists of two antiparallel β-sheets (a scaffold) connected by a loop region and stabilized by disulfide bonds (Fig. 2B). The loop region is more variable than the scaffold and plays a crucial role in the specificity of the binding to receptors [19]. The spatial structures of other family members have been determined via homology modeling.

Conserved cysteine residues are involved in the formation of disulfide bonds, whose number and arrangement affect the functional activity and conformation of the peptide. Thus, the ability to stimulate stomatal initiation was lost after cysteine residues had been replaced with serine in the EPFL9 molecule [19]. Conversely, variable regions can be responsible for the functional specificity of the peptides. Thus, EPF1/2 peptides act as negative regulators of stomatal development, whereas EPFL9 is a positive regulator [20]. The diversity in physiological responses are probably a result of structural differences in the loop region of these peptides [21].

Thus, replacing the EPF2 loop with the corresponding sequence from EPFL9 converted the peptide’s function from inhibition to promotion of stomatal development. Meanwhile, a chimeric peptide carrying the EPF2 loop and the EPFL9 scaffold exhibited an inhibitory activity [19]. The ERECTA family (ERf) kinases, which belong to the leucine-rich repeat receptor-like kinases (LRR-RLK) clade XIII, act as receptors for EPFL peptides. In Arabidopsis, this family includes the ERECTA (ER), ERECTA-LIKE 1 (ERL1), and ERECTA-LIKE 2 (ERL2) proteins. The combined signaling pathway involves the MAPK (mitogen-activated protein kinase) cascade, which consists of MAPKKK YODA, MKK4/5, and the terminal kinases MPK3/6 in Arabidopsis [27]. The peptide–receptor interaction depends on whether the receptor is part of a complex with LRR–RLP (leucine-rich repeat receptor-like protein) TMM (Too Many Mouths). Interestingly, EPF1/2 bind only to the ERf–TMM complex, while EPFL4 interacts with each of three ERf in the absence of TMM [21].

## PHYLOGENETIC DIVERSITY IN PLANTS

The EPF and EPFL peptides have been identified only in terrestrial plants, but they are not found in algae [28, 29]. This indicates that this peptide family evolved after plants had colonized the land and may have played an important role in their adaptation to terrestrial life. There is a hypothesis holding that the key genetic components ensuring the formation of the stomatal apparatus, including EPF/EPFL, originated at the early stages of the evolution of terrestrial plants [30].

Peptide sequences are conserved across different taxa: PpEPF1, a homolog of AtEPF1 and AtEPF2, was identified in moss Physcomitrium patens. Phylogenetic analysis shows that PpEPF1 is closer to AtEPF1 and AtEPF2 than AtEPFL9 [28]. This is rather interesting, since the stomatal apparatus of mosses differs from that of angiosperms, and yet their developmental mechanisms seem to be similar [31, 32]. In addition to PpEPF1, ten EPFL peptides have been identified in moss; their functions are still to be characterized [28]. In angiosperms, the genes encoding EPF/EPFL peptides are unevenly distributed across chromosomes, which may be a result of genetic duplication events [33, 34]

This peptide family in Arabidopsis is phylogenetically subdivided into four clades: EPF1–EPF2– EPFL7, EPFL9, EPFL1–3, and EPFL4–6–EPFL8 (Fig. 1) [28, 34]. These groups differ in both structure and putative functions. Thus, members of the EPF1–EPF2–EPFL7 clade carry four conserved disulfide bonds, one located in the loop region, whereas the peptides from the other clades carry three disulfide bonds. This feature affects the ability of the peptides to bind to receptor complexes [21, 28].

The EPFL9 peptide was found in all the studied vascular plants, from lycophytes (Selaginella moellendorffii) and gymnosperms to angiosperms [28]. However, it was not identified in moss P. patens, although a EPF1/EPF2 homolog is present in that plant. Notably, the emergence of EPFL9, which activates stomatal development, coincides with an abrupt rise in stomatal density on leaf surfaces in the Late Devonian period, when megaphylls – large leaves with a well-developed vascular system – evolved [28, 35].

The number of sequenced plant genomes has recently increased, thus substantially facilitating the search for and subsequent validation of homologs. The genomes of a large number of agricultural flowering plants have been analyzed using bioinformatic tools. A total of 132 EPF/EPFL genes have been identified in the four cotton plant genomes: 20 and 24 genes in diploid species, and 44 genes in each of the tetraploid species [34]. Fourteen genes have been identified in potato plants [36]; and 27 genes, in rapeseed [37]. Fifteen EPF/EPFL genes have been identified in the black cottonwood Populus trichocarpa [38], while 14 genes have been in the Euphrates poplar P. euphratica [33]. EPFL genes were also discovered in monocots: 12 genes were identified in rice, sorghum, and rye [39, 40, 41]; 18 genes, in maize [42]; and 35 genes, in wheat [43]. The considerable abundance of the EPF/EPFL genes across different evolutionary lineages of angiosperms and other plants underscores their importance in adaptation to terrestrial environments, while the functions of many recently identified homologs remain unclear, requiring further experimental research.

## STOMATAL INITIATION

**Fig. 3:**
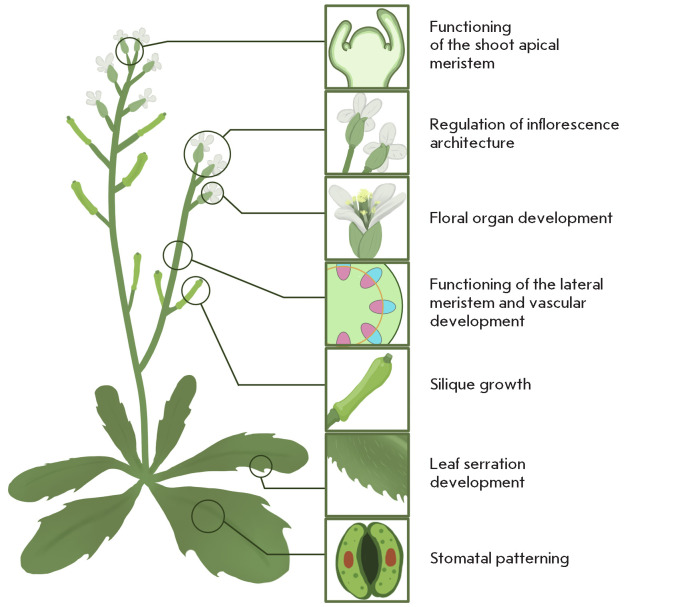
Morphogenetic processes regulated by peptides of the EPF/EPFL family in Arabidopsis thaliana

EPF/EPFL peptides are known to orchestrate a broad spectrum of morphogenetic programs; regulation of stomatal patterning was the first function of these peptides to be discovered (Fig. 3, Table 1) [10].

In Arabidopsis, EPF1 is expressed in young leaves; namely, in stomatal precursor cells. EPF1 overexpression reduces the stomatal density, while EPF1 knockout increases the stomatal density and clustering [10]. The EPF1 homolog, EPF2, also inhibits stomatal development: plants that overexpress the EPF2 gene are characterized by a reduced stomatal density, whereas EPF2-knockout plants demonstrate an increased stomatal density but do not form clusters [13]. Both peptides enforce the “one-cell spacing rule” dictating that at least one intervening nonstomatal epidermal cell should separate two stomata [10, 13, 14]. EPF2 is expressed in stomatal precursors earlier than EPF1.

**Table 1:** Functions of the EPF/EPFL peptides in A. thaliana

Peptide	Function	Reference
AtEPF1/2	Inhibition of stomatal formation	[10, 13, 15, 23, 44]
AtEPFL9 (STOMAGEN)	Stimulation of stomatal formation	[11, 15, 44, 45]
	Silique elongation	[46]
AtEPFL2	Leaf serration development	[47]
	Regular ovule spacing and increased ovule number per silique	[46]
AtEPFL1/2/4/6	Regulation of functioning of the apical meristem	[48, 49, 50, 51]
	Enhancement of pathogen resistance	[52]
	Elongation of inflorescences and pedicels	[53]
	Formation of a single megaspore mother cell	[54]
AtEPFL1–6	Envelopment of the nucellus by integuments	[55]
AtEPFL4–6	Stamen filament elongation due to cell proliferation	[56, 57]

Thus, EPF2 regulates the initiation of stomatal differentiation, while EPF1 controls further development [10, 14, 15]. Contrariwise, the EPFL9 peptide promotes stomatal development: EPFL9 overexpression increases stomatal density and causes clustering, whereas silencing of EPFL9 inhibits stomatal development [45]. Both the EPF1–2 and EPFL9 peptides have been shown to bind to the ER receptor; EPFL9 competitively displaces EPF1–2 from this complex [44]. The EPF1/2 peptides are expressed in stomatal cells, bind to ER and ERL1, and inhibit stomatal differentiation, whereas EPFL9 is expressed in mesophyll cells, competes with EPF2 for binding to ER, and promotes stomatal formation [15, 44]. Hence, EPF1/2 and EPFL9 act as antagonists in the stomatal density control [44].

The role of EPF peptides are best studied in Arabidopsis; however, their involvement in the regulation of stomatal development has been demonstrated for other plants as well. For example, overexpression of poplar PeEPF2, a homolog of AtEPF2, in AtEPF2 knockout Arabidopsis plants reduced the stomatal density on leaves and rescued the mutant phenotype [33]. Orthologs of AtEPF2 and AtEPFL9 involved in the regulation of stomatal development in Arabidopsis have been detected in the genomes of the monocots Triticum aestivum and Brachypodium distachyon [58]. These peptides also exert an opposing effect on stomatal development.

It has been demonstrated that the EPF/TMM/ ERECTA module is a rather ancient regulator of stomatal development: its components control stomatal patterning in early terrestrial plants, in moss P. patens in particular [59]. In P. patens, stomata form on the sporophyte; PpEPF1, a homolog of Arabidopsis EPF1/2, negatively regulates their development. However, PpEPF1 overexpression cannot restore the normal stomatal density in the Arabidopsis mutant epf2. Meanwhile, P. patens lacks an AtEPFL9 ortholog and AtEPFL9 overexpression does not affect its stomatal density, an indication that competitive regulation of stomatal patterning emerged at later stages of terrestrial plant evolution [59].

Hence, EPFL peptides are conserved and ancient regulators of stomatal development in terrestrial plants.

## FUNCTIONING OF THE SHOOT APICAL MERISTEM

The shoot apical meristem is a key structure that ensures the development of plant aerial organs. Its spatial organization, size, and activity are tightly regulated by a network of signaling cascades: EPFL peptides also participate in the process.

In A. thaliana, the EPFL1, EPFL2, EPFL4, and EPFL6 peptides are preferentially expressed in the periphery of the shoot apical meristem and within the boundary region between the meristem and leaf primordia [48]. Meanwhile, the ER, ERL1, and ERL2 receptors are active in the central zone of the meristem, suggesting that they are involved in the spatial regulation of meristem cell division and differentiation. The EPFL1/2/4/6 and ERf knockout mutants share a phenotype: a larger meristem, fewer leaf primordia, and a reduced overall plant biomass [48]. These data support the hypothesis that EPFL peptides and ER receptors are functionally redundant when regulating the size of the shoot apical meristem and initiating leaf growth [60].

EPFL2 knockout mutants exhibit disrupted symmetry and irregular organ spacing, as well as changes in the auxin maxima number in the shoot apical meristem [49]. That is consistent with the results of another study that reported that epfl2 mutants showed impaired shape of leaves and cotyledons due to change in auxin maxima number [50].

Furthermore, it has been demonstrated that treatment with synthetic EPFL4 and EPFL6 peptides ERfdependently limits the lateral growth of the meristem by downregulating the expression of the key apical meristem regulators CLV3 (CLAVATA3) and WUS (WUSCHEL) [51]. The interaction between these peptides and their receptors determines both the meristem size and its boundaries, thus contributing to the regulation of the number of initiated organs and ensuring normal plant development.

Hence, EPFL peptides play a pivotal role in the spatiotemporal regulation of the activity of the shoot apical meristem.

## REGULATION OF THE LATERAL MERISTEM AND VASCULAR DEVELOPMENT

Regulation of lateral meristems and vascular tissue initiation are the key processes responsible for the proper development of both vegetative and reproductive organs. The receptor kinases ER and ERL1 participate in the regulation of lateral meristems in the hypocotyls and inflorescence of Arabidopsis [61, 62, 63]. Thus, expression of the ER and ERL1 genes – but not ERL2 – was detected in the central cylinder of the hypocotyl [61]. In comparison with wild-type plants, er erl1 double mutants have thickened hypocotyls caused by excessive xylem development; this xylem has a higher proportion of cells with lignified cell walls [61]. In other words, ER and ERL1 prevent excessive xylem development in hypocotyls.

The ER and ERL1 kinases also regulate procambium development in inflorescence stems [62, 63]. The vascular bundle structure was impaired in er erl1 double mutants: the procambium layer was discontinuous, and direct contact between xylem and phloem frequently occurred. It has been demonstrated that ER and ERL1 are expressed in the xylem and phloem, phloem-specific expression of the ER gene being crucial for the regulation of the anatomical structure of the inflorescence stem [62]. It is hypothesized that the EPFL4 and EPFL6 peptides, which are expressed in the endodermis and bind to ER, are involved in this process. However, the epfl4 epfl6 double mutant does not seem to have a disrupted vascular bundle structure. Therefore, it still remains an open question which EPFL peptides are involved in the regulation of the lateral meristem function.

Hence, it has been demonstrated that the ER and ERL1 receptors – and presumably their ligands – participate in the regulation of the formation and function of lateral meristems, as well as vascular tissue initiation.

## DEVELOPMENT OF THE SERRATED LEAF MARGIN

Another role of EPFL peptides is the development of leaf margin serration [47]. In Arabidopsis, this process is regulated by the EPFL2 peptide, together with the ER and ERL1/2 receptors. EPFL2 knockout mutants, as well as ERf double mutants, have no serrated leaf margin. Moreover, the interaction between EPFL2 and each of the three ERf has been confirmed by co-immunoprecipitation [47]. The EPFL2 gene is expressed in growing leaves, except for the serrated tips and developing veins [47]. Interestingly, the ERL2 expression contrasts with that of EPFL2: it has been detected on the serrated tips and in the veins, while ER and ERL1 are expressed in the entire leaf blade. Hence, the EPFL2–ERf regulatory module suppresses the auxin response, confining it to a few cells on the tip of the developing serration.

## THE DEVELOPMENT OF REPRODUCTIVE ORGANS

Angiosperms have evolutionarily developed complex and diverse reproductive structures, with EPF/EPFL peptides playing a crucial role in the formation of these structures, from regulating the inflorescence architecture to seed formation.

EPFL4/6, and to a lesser extent EPFL1/2 together with ERf, stimulate the elongation of inflorescences and pedicels in A. thaliana [53]. EPFL4/6, which act as ER ligands, are expressed in endodermal cells, while the ER gene is expressed in the epidermis, phloem, and xylem. However, signal reception in the phloem is essential for the development of a normal inflorescence architecture, since ER expression under the phloem-specific SUC1 promoter restores the phenotype of er mutants. This effect has not been observed for ER expression under promoters active in the xylem and epidermis [53]. Hence, EPFL4/6 peptides are expressed in endodermal cells in plant inflorescences and transported to the phloem, where they bind to the ER and stimulate the growth of the inflorescence stem and pedicels [53]. Transcriptomic data demonstrate that many differentially expressed genes in A. thaliana er-2 and epfl4/6 mutants are components of the auxin and gibberellin response pathways. In particular, the expression of ARGOS, which promotes the growth of aerial organs [64], is suppressed, as well as the expression of the transcription factor WRKY15 [53].

The role played by EPFL peptides in the regulation of the inflorescence architecture has also been demonstrated in rice. Thus, OsEPFL5–9 regulate the panicle architecture and grain size. OsEPFL6–9 decrease the number of spikelets per panicle, while OsEPFL5 increase it, acting as an antagonist [65]. Further signaling in OsEPFL6–9 proceeds via the OsER1 receptor and the MAPK cascade comprising OsMKKK10–OsMKK4–OsMPK6 [65, 66].

Peptides belonging to the EPFL family control not only the overall development of inflorescences, but also the development of male and female reproductive organs and the resulting fruits. In Arabidopsis plants, EPFL4/5/6 promote stamen filament elongation by regulating cell proliferation [56, 57]. Impaired selfpollination and male sterility are observed in epfl4/5/6 triple mutants, since stamens become significantly shorter than the pistil [57]. At lower temperatures, self-pollination is already impaired for the mutant carrying a single epfl6 mutation [56]. ER mediates the elongation of both the stamens and the pistil [56].

EPFL1 in T. aestivum and EPFL6 in Brassica napus also appear to regulate the morphology of floral organs. Their overexpression in A. thaliana plants reduces the number of stamens and the stamen-to-pistil length ratio [67, 68].

EPFL peptides can also regulate A. thaliana silique development. EPFL9 recognized by ER promotes silique elongation, whereas EPFL2 expressed in interovule spaces increases the number of ovules per silique and ensures regular ovule spacing by interacting with the ERL2 and ERL1 receptors [46]. Interestingly, EPFL9 and EPFL2 may act as antagonists, since the EPFL9 expression under the EPFL2 promoter produce a phenotype similar to that of the epfl2 mutant [46].

EPFL1/2/4/6 also control the initial stages of female gametophyte development. These peptides are needed for differentiation of a single megaspore mother cell (MMC), preventing both the initiation of multiple MMCs and their absence [54]

At later stages of ovule development, EPFL1–6 ensure proper envelopment of the nucellus by integuments [55]. The EPFL1–6, ER, and ERL1/2 genes are expressed at different ovule developmental stages, while mutations in these genes disrupt integument formation. In this process, SERK1/2/3 function as coreceptors: the interaction between SERK and ERf kinases is enhanced in the presence of exogenous EPFL4/6 peptides [55].

EPF/EPFL peptides control the awn development, an important agricultural trait of rice. In wild rice (Oryza rufipogon) the EPFL1 gene is actively expressed in developing inflorescences and ensures the formation of longer awns and fewer grains per panicle [69]. Mutations altering the number of cysteine residues in OsEPFL1 were detected in most awnless cultivars of rice O. sativa, and introduction of the EPFL1 allele from African rice cultivar (O. glaberrima) leads to awned seeds in O. sativa ssp. japonica [69]. In the O. sativa ssp. aus cv. Kasalath, other EPF/EPFL genes are responsible for the awned phenotype: the osepfl1 single mutant retains awns, whereas the osepfl2 mutant is awnless and displays shorter grains, lower grain weights, and a decreasing number of cells along the longitudinal axis. OsEPF2, OsEPFL7, OsEPFL9, and OsEPFL10 also contribute to awn development. Both the OsEPFL1/GAD1/RAE2 and OsEPFL2/9/10 genes are believed to have undergone selection during rice domestication [39, 69].

The functions of EPFL peptides in the reproductive development of plants are extremely diverse. The members of this family orchestrate the inflorescence architecture, growth of floral organs, and proper formation of the female gametophyte.

## ABIOTIC STRESS

In recent years, multiple studies have concentrated on the identification of EPF/EPFL genes in various crop species. The promoter regions of these genes have been often found to contain the cis-regulatory elements associated with responses to stress factors and phytohormones [33, 34, 36, 40, 41]. Moreover, it has been experimentally verified that these factors regulate the expression of individual EPF/EPFL genes. This suggests that EPF/EPFL peptides may contribute to plant tolerance to environmental stress.

For example, EPFL8 expression is upregulated after treatment of maize plants with abscisic acid (ABA), methyl jasmonate, and salicylic acid, while expression of a number of other EPFL genes is downregulated under the same conditions [34]. Furthermore, water deficit can simultaneously alter the expression of several EPFL genes, indirectly demonstrating that they are possibly involved in the regulation of the drought response [34, 70]. Rye has both osmotic stress-induced and osmotic stress-repressed EPFL genes, as well as two heat-inducible EPFL genes [40]. A significant decline in the expression of seven EPF genes in rapeseed in response to salt stress was demonstrated in [37]. EPFL genes differentially expressed in response to osmotic stress have also been identified in sorghum, potato, poplar, and apple [33, 36, 41, 71].

EPF1/2 are known to inhibit stomatal formation in A. thaliana, while EPFL9 promotes it [10, 13, 45]. Stomatal density and transpiration intensity are responsible for the drought resistance of a plant. Comparison of the expression of EPF/EPFL genes in drought-tolerant and drought-sensitive apple (Malus domestica) cultivars has demonstrated that the expression of MdEPF2, an AtEPF2 ortholog, is more strongly induced by drought in the leaves of tolerant cultivar [71]. Treatment with abscisic acid (ABA), a key regulator of the osmotic stress response, also induces MdEPF2 expression. Tomato plants overexpressing MdEPF2 were shown to exhibit enhanced tolerance to osmotic stress. Under drought conditions, these plants were characterized by greater biomass, higher photosynthetic rates and relative water content, lower levels of malondialdehyde (MDA, a marker of oxidative stress) and hydrogen peroxide, as well as higher activity of antioxidant enzymes compared to that in wild-type plants [71]. The primary morphological effect of MdEPF2 overexpression consisted in a decline in stomatal density, which can be considered a key reason behind the greater osmotic stress tolerance observed in these plants.

The physiological role of the AtEPF2 ortholog, PdEPF2, identified in the poplar genome was studied previously [72]. Expression of PdEPF2 is induced by drought and ABA. Arabidopsis plants overexpressing PdEPF2 showed enhanced drought tolerance: their proline level and photosynthetic intensity were increased under osmotic stress conditions.

Four EPF/EPFL genes respond to drought in potato: EPF4 is downregulated, while the other three genes are upregulated [36]. Plants with either EPF4 knockdown or EPF4 overexpression were generated. Knockdown of this gene increased drought tolerance. Under drought stress these plants had a higher relative water content, proline level, and displayed activity of antioxidant enzymes (SOD, POD, and CAT), along with a lower MDA level than in wild-type plants. Conversely, the opposite effects were observed under drought conditions in plants overexpressing EPF4 [36]. Altered EPF4 expression affected the stomatal density, which was lower in EPF4 knockdown plants and higher in plants overexpressing EPF4. The negative role of EPF4 in the regulation of the osmotic stress response can possibly be associated with its effect on stomatal formation.

Taken together genomic and physiological data obtained for various agricultural crops, it can be concluded that EPF/EPFL peptides are potentially involved in plant responses to abiotic stresses, primarily to drought. Regulation of stomatal density and transpiration are the most frequently proposed mechanisms of action for these peptides; however, other mechanisms cannot be ruled out. Different members of this family can play both a positive and negative regulatory role, which underscores the functional diversity of EPF/EPFL peptides and suggests that further research into their specific functions across different physiological contexts is needed.

## BIOTIC STRESS

Differential expression of various EPF/EPFL members was shown in several plant species upon infection by phytopathogenic fungi. Thus, infection of moss P. patens with the pathogenic fungus Botrytis cinerea significantly downregulates expression of the six genes encoding the predicted EPFL peptides [73]. It was demonstrated that the expression of the EPFL1–6 and EPFL9 genes in A. thaliana increases after inoculation with Sclerotinia sclerotiorum, while the expression of other members of the EPF/EPFL family remains unaltered [52]. Meanwhile, biotic stress appeared to have different effects on the expression of the EPF/EPFL genes in tomato Solanum lycopersicum plants. Thus, infection with the phytopathogen Fusarium oxysporum f. sp. lycopersici induces the expression of SlEPF7 and decreases the expression of SlEPF1/5. Treatment with elicitors from a non-pathogenic for tomato F. sambicinum strain increases SlEPF6/7 expression and decreases that of SlEPF3/5 [74].

Simultaneous changes in the expression level of several EPF/EPFL genes upon interaction with phytopathogens suggest that peptides belonging to this family can coordinately regulate plant defense mechanisms. Thus, the growth of S. sclerotiorum and H_2_O_2_ generation were shown to increase significantly in Arabidopsis epfl1,2,4,6 multiple mutants, whereas single mutants did not differ from wild-type plants [52]. Furthermore, pathogen-induced expression of the genes belonging to the YODA DOWNSTREAM (YDD) group was significantly reduced in the epfl1,2,4,6 mutants. YDD is a group of genes positively regulated in constitutively active YODA mutants [52]. On the other hand, inducible EPF1/2 expression in A. thaliana did not enhance plant resistance to the necrotrophic fungus Plectosphaerella cucumerina [75]. Many pathogens are known to penetrate into plant tissues through stomata; therefore, the weakened resistance of ERf mutants can plausibly be attributed to the increased stomatal density. Thus, treatment with EPFL9 increases the stomatal density and exacerbates the symptoms of infection [76].

Hence, data on the involvement of EPF/EPFL peptides in the regulation of the biotic stress response are extremely sparse. Meanwhile, it has been repeatedly demonstrated that receptors and components of the EPF/EPFL peptide signaling pathway are involved in ensuring phytopathogen resistance. Thus, er mutants were characterized by reduced resistance to the bacterium Ralstonia solanacearum [77], oomycete Pythium irregulare [78], as well as the pathogenic fungi Verticillium longisporum [79], S. sclerotiorum [80], and P. cucumerina [81, 82]. Additional knockout of the ERL1/2 and TMM genes exacerbated infection symptoms [75, 80].

However, the reduced resistance to R. solanacearum after inoculation through damaged roots [77] indicates that the susceptibility of er mutants may be caused not only by the increased stomatal density but also by an impaired defense response. This is further supported by the downregulated expression of the pathogen-inducible genes WRKY33, WRKY53, CYP79B2, and CYP81F2 in er, bak1, and er bak1 mutants [75].

Meanwhile, the activity of ER was shown to have no effect on the expression of the genes induced by flg22, a 22-amino acid flagellin-derived epitope [75]. Furthermore, er mutants were no less resistant to infection by B. cinerea, F. oxysporum f. sp. conglutinans, and Peronospora parasitica than wild-type plants [81]. Therefore, ER is not always required for pathogen resistance. This can be associated with the functional redundancy of EPFL receptors.

ER regulates the Arabidopsis response to S. sclerotiorum infection via affecting binding between the WRKY33 transcription factor and promoters of the YDD genes [80]. This process involves the chromatin remodeling complex SWR1 and the ER–MPK6– WRKY33 regulatory module. SWR1 promotes the binding of the W-box transcription factor WRKY33 to promoters and activates expression of the YDD genes, which are necessary for resistance to S. sclerotiorum infection [80, 83].

Since EPF/EPFL peptides are primarily known as regulators of stomatal development, their role in stress adaptation is often attributed to their impact on stomatal density. However, the role of this peptide family under stress conditions appears to be broader and needs further investigation.

## CONCLUSIONS

Despite significant progress in understanding EPF/ EPFL peptides functions, knowledge gaps still remain. Thus, the vast majority of functional studies on EPF/EPFL have been conducted on the model plant A. thaliana. Furthermore, although homologs of the EPF/EPFL genes have been identified across different groups of angiosperms, their function need more comprehensive investigation. This issue is particularly relevant in the context of the plant phylogenetic diversity, since the results obtained for Arabidopsis may not fully represent the range of biological functions of EPFL peptides in other plant species.

Additional challenges arise from the functional redundancy of these peptides: multiple EPF/EPFL family members can partially compensate for each other, thus complicating the assessment of individual contributions. So, much of the research analyzes receptor mutants, which are also partially redundant, but their number is significantly smaller

It has been demonstrated so far that EPFL expression can be altered in response to biotic and abiotic stresses; however, the association between peptidemediated regulation and plant adaptive responses still needs to be fully elucidated.

Modulating the activity of EPFL peptides and their receptors may be used to optimize morphogenesis, enhance stress tolerance, and, therefore, improve cultivated crops.

## Abbreviations

EPF: epidermal patterning factor
EPFL: epidermal patterning factor-like
ABA: abscisic acid
MAPK: mitogen-activated protein kinase
MDA: malondialdehyde
MMC: megaspore mother cell
